# Azacytidine plus olaparib for relapsed acute myeloid leukaemia, ineligible for intensive chemotherapy, diagnosed with a synchronous malignancy

**DOI:** 10.1111/jcmm.16513

**Published:** 2021-06-16

**Authors:** Sabina Iluta, Sergiu Pasca, Grigore Gafencu, Ancuta Jurj, Andreea Terec, Patric Teodorescu, Cristina Selicean, Ciprian Jitaru, Alexandra Preda, Diana Cenariu, Catalin Constantinescu, Maria Iordache, Bogdan Tigu, Raluca Munteanu, Richard Feder, Delia Dima, Mihnea Zdrenghea, Diana Gulei, Tudor‐Eliade Ciuleanu, Ciprian Tomuleasa

**Affiliations:** ^1^ Department of Hematology Iuliu Hatieganu University of Medicine and Pharmacy Cluj Napoca Cluj Napoca Romania; ^2^ Department of Hematology Ion Chiricuta Clinical Cancer Center Cluj Napoca Cluj Napoca Romania; ^3^ Medfuture Research Center for Advanced Medicine Iuliu Hatieganu University of Medicine and Pharmacy Cluj Napoca Cluj Napoca Romania; ^4^ MRC Molecular Haematology Unit ‐ The MRC Weatherall Institute of Molecular Medicine University of Oxford Oxford UK; ^5^ Research Center for Functional Genomics and Translational Medicine Iuliu Hatieganu University of Medicine and Pharmacy Cluj Napoca Cluj Napoca Romania; ^6^ Department of Leukemia The Sidney Kimmel Comprehensive Cancer Center Johns Hopkins University School of Medicine Baltimore US; ^7^ Department of Hematology Victor Babes University of Medicine and Pharmacy Timisoara Romania; ^8^ Department of Medical Oncology Iuliu Hatieganu University of Medicine and Pharmacy Cluj Napoca Cluj Napoca Romania; ^9^ Department of Chemotherapy Ion Chiricuta Clinical Cancer Center Cluj Napoca Romania

**Keywords:** clinical scenario, combination chemotherapy, hypomethylating agents, refractory acute myeloid leukaemia, synthetic lethality

## Abstract

Patients with relapsed/refractory acute myeloid leukaemia (AML), ineligible for intensive chemotherapy and allogeneic stem cell transplantation, have a dismal prognosis. For such cases, hypomethylating agents are a viable alternative, but with limited success. Combination chemotherapy using a hypomethylating agent plus another drug would potentially bring forward new alternatives. In the present manuscript, we present the cell and molecular background for a clinical scenario of a 44‐year‐old patient, diagnosed with high‐grade serous ovarian carcinoma, diagnosed, and treated with a synchronous AML. Once the ovarian carcinoma relapsed, maintenance treatment with olaparib was initiated. Concomitantly, the bone marrow aspirate showed 30% myeloid blasts, consistent with a relapse of the underlying haematological disease. Azacytidine 75 mg/m^2^ treatment was started for seven days. The patient was administered two regimens of azacytidine monotherapy, additional to the olaparib‐based maintenance therapy. After the second treatment, the patient presented with leucocytosis and 94% myeloid blasts on the bone marrow smear. Later, the patient unfortunately died. Following this clinical scenario, we reproduced in vitro the combination chemotherapy of azacytidine plus olaparib, to accurately assess the basic mechanisms of leukaemia progression, and resistance to treatment. Combination chemotherapy with drugs that theoretically target both malignancies might potentially be of use. Still, further research, both pre‐clinical and clinical, is needed to accurately assess such cases.

## 1 INTRODUCTION

Acute myeloid leukaemia (AML) is a malignancy of the myeloid hematopoiesis, characterized by the accumulation of accumulating genetic aberrations.^1^, ^2^, ^3^, ^4^ Progress in next‐generation sequencing (NGS) has successfully risk‐classified AML in accordance with specific gene mutations or associations of gene mutations.^5^, ^6^, ^7^ Understanding the genetic background of AML yields new therapies that could potentially involve the use of new alternatives.^8^, ^9^

In the current manuscript, we present the clinical scenario of a 44‐year‐old woman, diagnosed with concomitant relapsed ovarian carcinoma and relapsed AML. She received therapy with the poly‐ADP ribose polymerase (PARP) inhibitor olaparib for the ovarian cancer. Not eligible for intensive chemotherapy, treatment with azacytidine was subsequently initiated. After two cycles of treatment, the patient succumbed due to infections. Following this therapeutic failure, we aimed to assess the cellular mechanisms of disease progression in vitro. Thus, we investigated the effects of olaparib that causes synthetic apoptosis in cancers with homologous recombination deficiencies (HRD),^10^ in combination with daunorubicin (ODC) or azacytidine (AZA), on two AML cell lines. These settings simulated both the first‐line chemotherapy for AML and chemotherapy‐refractory AML. The first cell line, OCI/AML3, is characterized by the occurrent mutations in both nucleophosmin (NPM1), a gene involved in DNA single‐strand break repairs,^11^ and DNA methyltransferase 3 alpha (DNMT3A), a gene involved in resistance to chemotherapy‐induced DNA damage.^12^ Both mutations are common and present a significant impact in the prognosis of AML patients.^5^ The second cell line, THP‐1, has mutations and deletions in PTEN, MLL‐AF9, MLLT3, TP73 and CDKN2A/B.^13^, ^14^ The genetic landscape of THP‐1 cell line makes it susceptible to the effects of olaparib, particularly through the presence of a partial deletion in PTEN gene.^9^

Consequently, we conducted a set of in vitro assays to establish whether OCI/AML3 is affected by ODC in a similar fashion as THP‐1 cells, in comparison with the effects generated by the standard cytarabine‐daunorubicin regimen (CDR) (therapy given in first line for AML), as well as in comparison with AZA‐based chemotherapy (therapy given to patients ineligible for intensive chemotherapy), thus simulating all the possible clinical scenarios in which a PARP inhibitor might be used in the clinic, in order to properly explain the basic mechanisms of disease progression and resistance to chemotherapy, as THP‐1 is PTEN mutant and thus susceptible to olaparib treatment.

## 2 CLINICAL SCENARIO

In the present manuscript, we present the case of a 44‐year‐old young woman which was diagnosed with high‐grade serous carcinoma of ovarian origin, pT3cNxMx FIGO III C, radically operated in 2016 and treated with adjuvant chemotherapy. Following routine medical analysis, bicytopenia was diagnosed, with marked leukocytosis and 95% blasts on the bone marrow smear. Diagnosis of AML was conclusive, both on the myelogram and flow cytometry. In the bone marrow, a percentage of 80% peroxidase‐positive blasts was detected. The immunophenotype was CD34+, HLA DR+, CD117+, CD45 low, CD117+, CD34−, HLA‐DR‐, CD13+, CD11b−, CD16−, CD10−, CD33+, CD64−, CD35−, CD300e−, CD14−, CD7−, CD19−, CD15−, CD22−, NG2‐, CD38+, TdT−, CD56 heterogeneous −/+, Cd71 heterogenous +/−, CD36−, CD105−, 1% monocyte mature CD300e+, CD64+, 2% promonocyte CD14+ CD64+, 1% monoblasts CD14− CD64+ CD117− and 1% monoblastic CD117+.

Molecular biology was negative for the FLT3 mutation, but positive for NPM1. Standard chemotherapy (‘3 + 7’) regimen was performed. At the end of the induction treatment, a control myelogram was performed, which showed a high percentage of blasts (40%). For the refractory AML, second‐line treatment was administered–the FLAG‐Etoposide regimen.^15^ After the second line of treatment, the AML was in complete remission (CR1), with 3%‐4% blasts on the control myelogram. Seven months after CR1, the patient presents with relapsed ovarian carcinoma, for which second‐line chemotherapy treatment with paclitaxel and carboplatin was started, after which the remission of ovarian carcinoma was obtained.^16^, ^17^ The mutational status of BRCA was positive, and subsequent maintenance treatment with olaparib is initiated.^18^, ^19^, ^20^ Seven more months after obtaining the second remission (CR2) of the ovarian carcinoma, on maintenance treatment with olaparib, the complete blood count (CBC) showed pancytopenia and the presence of 4% blasts on blood smear. The bone marrow aspirate showed 30% myeloid blasts, with relapsed AML. As the patient was not eligible for intensive chemotherapy, due to the altered physical status, the therapeutic options were now chemotherapy with azacytidine (AZA) monotherapy, decitabine monotherapy or low‐dose cytarabine.^21^, ^22^, ^23^ Taking into consideration the altered physical status of the patients, azacytidine 75 mg/m^2^ treatment was started for seven days. Taking into consideration that olaparib is currently under investigation for relapsed/refractory(R/R) AML in 2 clinical trials (Table 1), as well as considering that olaparib is currently used for the maintenance therapy for ovarian adenocarcinoma, the therapeutics committee decided to keep both olaparib and azacytidine therapy.

**TABLE 1:** Clinical trials investigating the role of olaparib in R/R AML

Clinical trial name	Clinical trial identifier	Recruitment status	Phase	Coordinating institution
Using the Anticancer Drug Olaparib to Treat Relapsed/Refractory Acute Myeloid Leukemia or Myelodysplastic Syndrome with an Isocitrate Dehydrogenase (IDH) Mutation	NCT03953898	Recruiting	II	Yale University Cancer Center, USA
A Personalized Medicine Study for Patients with Advanced Cancer of the Breast, Prostate, Pancreas or Those With Refractory Acute Myelogenous Leukemia (SMMART)	NCT03878524	Recruiting	II	Oregon Health and Science University, Knight Cancer Institute, USA

The patient was thus given three cycles of azacytidine plus olaparib combination chemotherapy. After the third cycle, the patient presented with leucocytosis and 94% myeloid blasts in the bone marrow. Later, the patient unfortunately died. Following this clinical scenario, we reproduced in vitro the combination of chemotherapy agents, to properly understand the basic mechanisms of leukaemia progression and resistance to chemotherapy.

## 3 MATERIALS AND METHODS

### 3.1 Cell Culture

Both cell lines were cultured in vitro to assess drug treatment, by plating the OCI/AML3 (DSMZ–Deutsche Sammlung von Mikroorganismen und Zellkulturen GmbH–German Collection of Microorganisms and Cell Cultures) and THP1 (ATCC–American Type Culture Collection) cells in 96‐well plates, at 10^4^ cells/200 µL/well in 2 types of media: 80% alpha‐MEM (Invitrogen) with 20% foetal bovine serum (FBS) (Invitrogen) for OCI/AML3 or RPMI1640 (Invitrogen) with 10% FBS, 2 mM L‐glutamine for THP1, and then treating them for 48 hour with 37.5 µM olaparib (Selleckchem), 100 µM cytarabine (Sigma‐Aldrich), 1.4 µM daunorubicin (Sigma‐Aldrich) and 10 µM azacytidine (Sigma‐Aldrich), either alone or in combination. Cells were cultured in an incubator at 37°C and 5% CO_2_, as previously described.^24^, ^25^, ^26^ Cell proliferation was evaluated by using the CellTiter 96® AQueous Non‐Radioactive Cell Proliferation Assay (Promega) and analysed with BioTek Synergy H1 Hybrid Multi‐Mode Reader (BioTek Instruments). All reagents and compounds were purchased from Invitrogen and had a 99.9% purity. The in vitro experiments were carried out after the approval of the Ethics Committee of the Iuliu Hatieganu University of Medicine and Pharmacy in Cluj Napoca.

### 3.2 Cell cycle assessment

For assessing cell cycle arrest, flow cytometry was used after 48h of treatment, as described by Esposito et al^27^ DNA double break (DSB) levels were assessed by quantifying the phosphoSer139 γH2AX foci, an event associated with DSB using a flow cytometry after a 48 hour and 72 hour of treatment, as described by Redon et al.^28^

### 3.3 qRT‐PCR analysis

qRT‐PCR was performed to analyse the gene expression in relation to previously described drug combinations on two AML cell lines. RNA isolation was performed by using TriReagent^TM^ Solution. Total RNA obtained was quantified with NanoDrop 2000®. Prior to performing reverse transcription, RNA samples underwent DNase treatment (TURBO DNase Free Kit, Invitrogen). 800 ng of total RNA was used for cDNA reaction with SuperScript™ III Reverse Transcriptase Kit. Random hexamer primers were used for the reaction. qRT‐PCR was carried out using SYBR Select Master Mix. Primers for ATM, RAD51, LIG3 and 4, PARP1, PTEN and B2 M (as internal normalizer) were custom made. Analysis was carried out on three separate biological experiments with ViiA 7® Real Time PCR System. All instruments and reagents were purchased from Thermo Fisher Scientific with the above‐mentioned exceptions. To calculate the relative mRNA expression, 2^−ΔΔCt^ method was used. We analysed all the data using R. All the experiments were carried out in triplicate and represented as boxplot. In data analysis, the visualizing package is ggplot2. Results were considered significant for *P* values ≤ .05, as previously described.^29^

### 3.4 Pre‐clinical murine testing of azacytidine plus olaparib for AML

Eight‐week‐old male athymic nude mice purchased from Charles River Laboratories were used in the study. The animals were housed in IVC2‐SM‐56‐IIL rack system (Acellabor) with individual ventilated cages supplied with HEPA‐filtered air (II L Cages) with ad libitum access to autoclaved water and pelleted feed. The bedding was also autoclaved according to the standard programme. The animals were maintained in the authorized animal facility from Medfuture Research Center for Advanced Medicine–Iuliu Hatieganu University of Medicine and Pharmacy, Cluj‐Napoca at a standard temperature of 22°C ± 2°C and a relative humidity of 55% ± 10%, in a 12:12 hour light: dark cycle. All experimental protocols were approved by the Ethics Committee of Iuliu Hatieganu University of Medicine and Pharmacy and were conducted in accordance with the EU Directive 63/2010. Before entering into the experiments, the animals were tagged with metallic ear tags and separated from the rest. The mice were injected into the knee joint with 5 × 10^6^ AML luciferase‐positive (AML‐Luc), cells cultured as previously mentioned (procedure developed under gas anaesthesia). The development of the tumour was followed for 20 days macroscopically, where at day 20, the installation of the xenograft was confirmed with IVIS SPECTRUM–IVIS Imaging System (Perkin Elmer) via the bioluminescent reporter optimized for in vivo imaging–RediJect D‐Luciferin (XenoLight, Perkin Elmer). The animals with uniform tumour patterns were dividend in three treatment groups: a (Control group), which received 200 µL of PBS for 5 days consecutively, b (5‐azacytidine group), which received 2.5 mg/kg 5‐azacytidine 5 days consecutively and c (5‐azacytidine plus olaparib group), which received 2.5 mg/kg 5‐azacytidine plus 50 mg/kg olaparib for 5 days consecutively. The doses were calculated according to Food and Drug Administration (FDA) guidelines for human dose conversion into animal equivalent dose (for mice, the equivalent human dose was multiplied by 12.3). 5‐azacytidine and olaparib were dissolved in PBS and injected intraperitoneally (same administration route for control group). Stock solution of 5‐azacytidine was initially prepared in DMSO and further diluted with PBS. Working solutions were prepared for each mouse and stored separately at −20°C in order to avoid repeated freeze‐thaw cycles. Considering the novelty of the experiment and the aggressiveness of the pathology, we decided to conduct first a pilot study and we included one mice/group (the ones with uniform tumour distribution), remaining to continue the study on larger cohorts. After 5 days of treatment (day 26), the efficiency of the therapeutics was assessed via bioluminescent imaging as stated before. Animals were weighed at the beginning of the experiment before treatment initiation and after treatment. Bioluminescent images were processed using Living Image®4.5.2 Software. The same software was used to automatically measure the signal intensity within the region of interest (ROI) using the automatic Contour tool.

## 4 RESULTS

**FIGURE 1:**
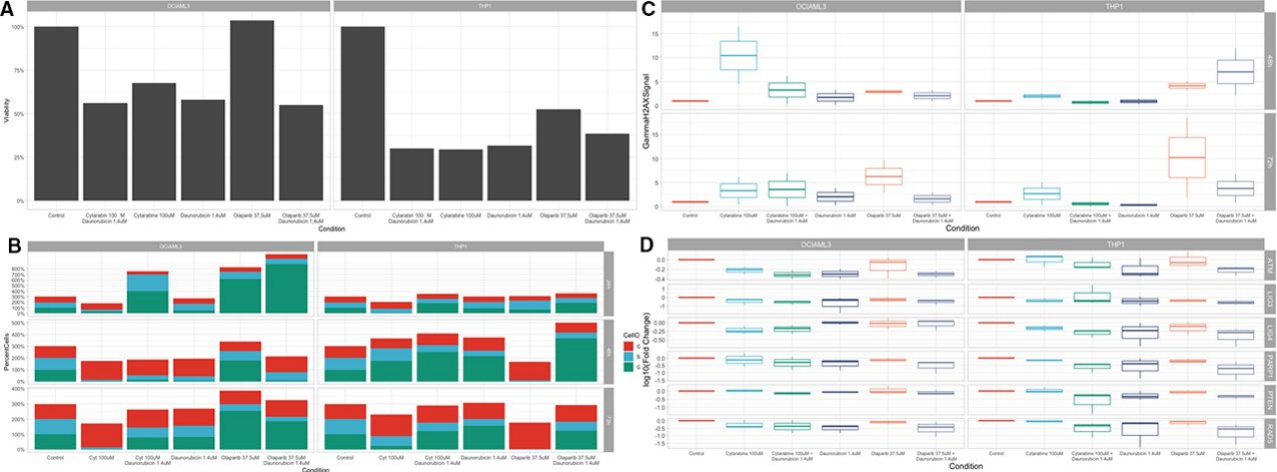
Pre‐clinical in vitro assessment of olaparib plus classic chemotherapy for AML

Cytarabine and daunorubicin are used in first‐line chemotherapy for AML, to achieve remission before an allogeneic stem cell transplantation.^30^ From the standpoint of the effects of ODC and CDR on OCI/AML3 and THP‐1 blast proliferation, ODC proved to be as potent as CDR in decreasing the viability of treated cells that was compared between cell lines (Figure 1A–OCI/AML3: 54.9% vs 56.1%, *P* = .999; THP1:38.5% vs 30.1%, *P* = .982). Statistical analysis for Figure 1C is shown in Supplementary Table S1. Statistical analysis for Figure 1D using ANOVA is shown in Supplementary Table S2, whereas using pairwise *t* test is shown in Supplementary Table S3.

**FIGURE 2:**
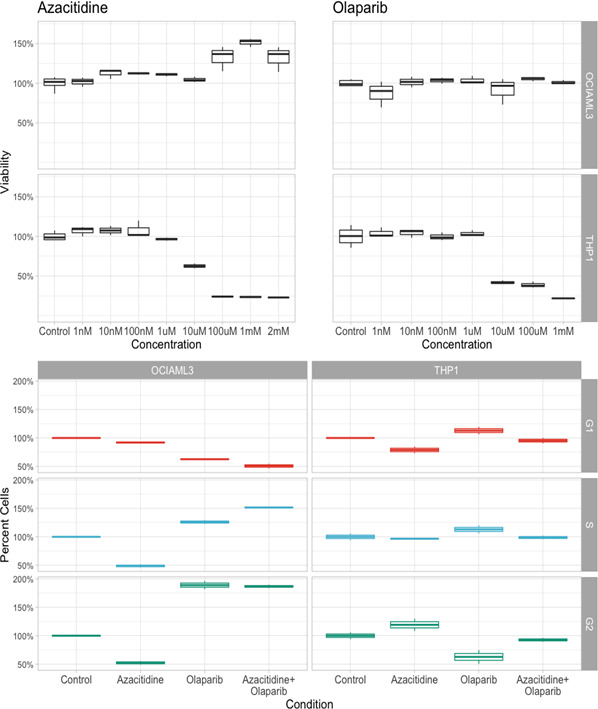
Cell cycle analysis of AML cells, following therapy with azacytidine, olaparib and its combination

Moreover, regarding the effects of ODC and CDR on the cell cycle stage of OCI/AML3 and THP‐1 blasts, ODC manages to induce similar effects in magnitude to the ones induced by CDR when we compared the percentage of cells in G1, S and G2‐M and cell cycle stage, regardless of cell line (Figure 2B). Still, for OCI/AML3 cells, ODC increases the percentage of cells in G2‐M, with 2.32% when compared to 4.16% for CDC (*P* > .05). Further on, following the quantification of the phosphoSer139 γH2AX foci via a flow cytometry‐based method at 48 hours and 72 hours time‐points. At 48 hours, there is no significant difference in the induction of DSB by ODC or CDR regardless of the treated cell line. But after 72 hours, statistically significant differences between the efficacy of inducing DSB by ODC and CDR are reported, at least for THP1 cell line (Figure 2C, OCI/AML3: 123% *vs*. 122%; THP‐1:115% *vs*. 133%, *P* = .0211). Statistical analysis for Figure 2A using ANOVA is shown in Supplementary Table S4, whereas using pairwise *t* test is shown in Supplementary Table S5. Statistical analysis for Figure 2B is shown in Supplementary Table S6.

In order to assess at functional level whether the high rate of response and similarities observed in both therapeutic approaches are in concordance with the gene expression, we performed qRT‐PCR and evaluated the expression of genes located downstream of the PARP signalling pathway.^31^, ^32^, ^33^, ^34^, ^35^ The results indicate that both ODC and CDR triggered after 48 hour comparable transcript levels of genes related to the DNA repair system. All the genes evaluated were found to display certain levels of down‐regulation (Figure 1D). Levels lower than 50% of the control for the OCI‐AML3 cell line were observed for two genes, ATM and LIG3, for both therapeutic approaches, but a 50% decrease in RAD51 and PARP1 expression was observed only for ODC‐treated cells.

A similar pattern, but more pronounced, was observed in THP‐1 for both ODC and CDR, where five genes, RAD51, PARP1, PTEN, LIG3 and LIG4, displayed a lower than 50% of the control down‐regulation. When comparing the gene expression differences for cells treated with OCD to the ones treated with CDR, we concluded that in OCI‐AML3 cell line four of the genes assessed (ATM, PTEN, LIG3 and LIG4) was found to be up‐regulated, and two down‐regulated (RAD51 and PARP). Out of the up‐regulated genes only LIG4 displayed a 2‐fold up‐regulation. When the same analysis was performed for THP‐1 cells, experimental data showed that all the genes were down‐regulated.

When looking at therapy with AZA, alone or in combination with olaparib, in vitro cell proliferation assays showed that at 48 hours post‐treatment, neither AZA alone, not in combination with olaparib had any effect on OCI‐AML3 cells. Moreover, cell proliferation even increased for the cells treated with these drugs (Figure 2A). Still, these drugs had in vitro inhibitory effects on THP1 cells. This is paradoxical, as OCI‐AML3 cells are NPM‐positive,^36^ this being the exact biological background of our patient: NPM‐positive/FLT3‐negative. THP1 cells harbour the PTEN mutation,^37^ with a negative prognosis in comparison with NPM‐positive myeloid leukaemia cells.^38^, ^39^, ^40^ This may be the ‘target’ population for combination chemotherapy of azacytidine plus olaparib.

Cell cycle analysis confirms this. PTEN‐positive cells (THP1 cell line) are scarcely affected by either of the drugs, with all cells being distributed almost equally in G1, S and G2 phases. Nevertheless, when analysing NPM1‐positive cells (OCI‐AML3 cells), most cells were in G2 phase, in concordance with the proliferating assay (Figure 2B).

**FIGURE 3:**
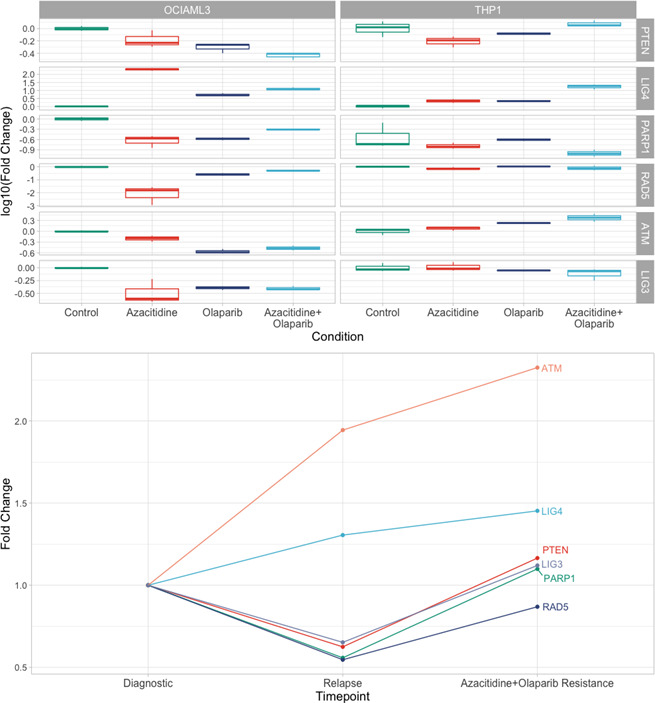
RT‐PCR assessment for cells treated with azacytidine, olaparib and olaparib plus azacytidine

The RT‐PCR analysis showed a down‐regulation of PARP1 and other genes associated with DNA damage repair, as are PTEN, ATM or LIG family genes, for the NPM1‐positive cells (OCI‐AML3 cells). These cells, following therapy with olaparib or olaparib plus AZA, behave totally different from PTEN‐positive cells (THP1 cells) (Figure 3A). The mutational profile of NPM1‐positive cells is the same as cells isolated from the bone marrow aspirate of our previously described case, with genes associated with DNA damage repair being down‐regulated from diagnosis to relapse. Once olaparib plus AZA was introduced, following relapse, the same genes were up‐regulated, consisting with patient leukaemia progression and resistance to therapy (Figure 3B).

**FIGURE 4:**
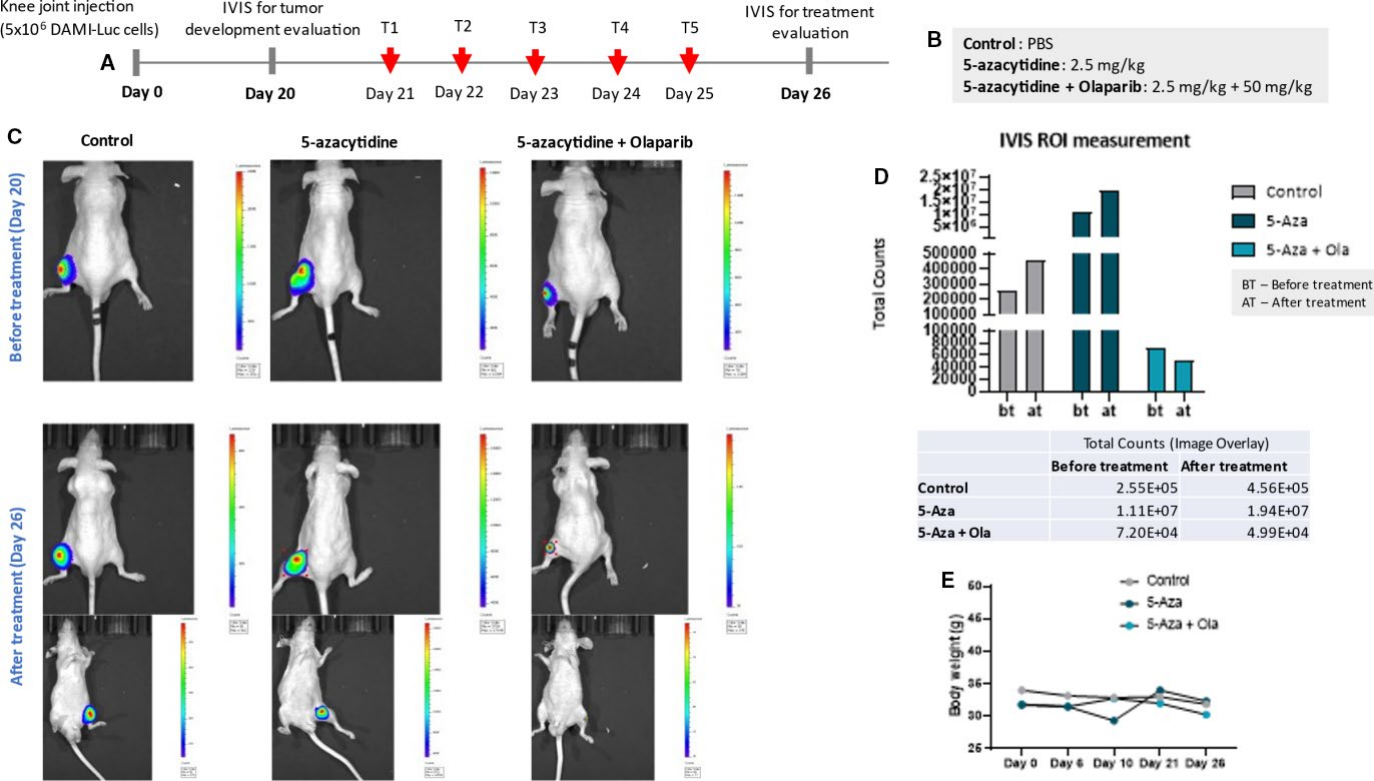
Pilot in vivo study for investigation of 5‐azacytidine plus olaparib combination chemotherapy efficiency for treatment of AML. (A). Experimental protocol and treatment scheme; (B). treatment groups and dosage according to FDA guidelines for human dose conversion into animal equivalents; (C). in vivo bioluminescent imaging of xenograft mice before and after treatment using IVIS Imaging System (Perkin Elmer) and bioluminescent reporter optimized for in vivo imaging; (D). Automatic ROI measurement of tumour signal intensity before and after treatment (Control, 5‐Aza, 5‐Aza +Ola); (E). mice bodyweight measurement (g) before treatment (Day 0, Day 6, Day 10 and Day 21) and after treatment (Day 26)

In order to investigate the efficiency of 5‐azacytidine plus olaparib combination chemotherapy for the treatment of AML, we developed an experimental mouse model of the disease by injecting AML‐Luc cells into the cartilage of the mice knee joint. We let the tumours to develop for 20 days to mimic an advanced form of the disease. Considering the highly experimental character of the protocol, we decided to conduct for the moment a pilot study for proof of principle and include only three mice with the most uniform tumour distribution in the leg as detected through bioluminescent imaging. The treatment protocol was followed for 5 consecutive days according to Figure 4A and consisted of 5‐azacytidine administrated alone or 5‐azacytidine given in combination with olaparib (Figure 4B). The second bioluminescent imaging exposure after the completion of the treatment showed that the mice from the control group had an progressive evolution of the malignant mass, while 5‐azacytidine managed to control to some extent the development of the AML‐Luc cells, delaying the tumour spread (Figure 4C). For the cohort with 5‐azacytidine plus olaparib combination chemotherapy, we show a decrease in the tumour formation, results confirmed also by automatic ROI measurement of the bioluminescent signal intensity (Figure 4D). No significant changes in the bodyweight of the mice or other adverse side effects were observed during the experiments, suggesting that the treatment was well tolerated (Figure 4E). Considering the aggressive phenotype of AML, where the survival is very poor even the best available therapeutic option, these results can translate in a significant improvement in the clinical management of the patients. Further studies on larger animal cohorts have to be put in place in order to predict the efficiency of the treatment combination for a potential phase I clinical trial.

## 5 DISCUSSION

For the potential first‐line clinical scenario, with AML treated with intensive chemotherapy, the biological effects inflicted by ODC on the blast cell proliferation, cell cycle and DNA damage levels proved to be similar to the ones induced by the CDR, regardless of the cell lines tested. Our results come to reinforce the data that show that THP1 is susceptible to the action of PARP inhibitors in conjunction with anthracyclines due to its MLL‐AF9 mutation.^41^ Thus, we establish that so is NPM1‐positive mutated AML in an in vitro setting. This biological scenario cannot only be attributed to increased amounts of DSB, as measured by the increase of phosphoSer139 γH2AX foci, but probably also on the effects that ODC had on the expression profile of RAD51 and PARP1 genes on both cell lines. Inhibition of PARP1 will delay the onset of ROS‐induced autophagy.^42^ Blunting the autophagic processes influences blast survival, particularly in PI3K/Akt/mTORC1 pathway deficient AML.^43^ Our tentative explanation for the effects that ODC generated on the selected AML cell lines in vitro implies the exciting hypothesis that combining PARP inhibitors and anthracyclines can capitalize on two defective apparatuses in AML: DNA repair, autophagy or possible cell differentiation by olaparib. This is of potential clinical impact, as it can be a viable therapeutic option, easing the side effect burden of intensive chemotherapy for AML, by potentially substituting cytarabine with olaparib in treating patients with NPM1‐DNMT3A mutated AML.

Still, when looking at the R/R AML scenario, for patients unfit or ineligible for intensive chemotherapy, the data show a totally different story. Adding olaparib to AZA has little or no effect for NPM1‐positive cells, as shown by in vitro cell proliferation assays in Figure 2A and backed up by cell cycle analysis and RT‐PCR of the genes involved in DNA repair (Figure 2B). This is consistent with the clinical evolution of our patient, who had a FLT3‐negative/NPM1‐positive mutational status.

PARP1 gene expression is also linked to DNA methylation, with important clinical impact in gynaecological malignancies, especially cancers that are BRCA‐mutated.^44^, ^45^, ^46^ Kondrashova et al have shown that methylation of the BRCA1 copies is linked to response to a PARP inhibitor. In a recent analysis of the clinical methylation, combined with expression data from the Cancer Genome Atlas Program (TCGA) cohort on adult AML, changes in the methylome have been linked with clinical prognosis, thus presenting the hypothesis that the effectiveness of PARP inhibition as an AML therapeutic agent to be linked to a specific AML methylome.^47^, ^48^ Nevertheless, further studies are required to test this hypothesis before we can move on and test the combination chemotherapy of azacytidine plus olaparib in a phase I clinical trial.

## 6 CONCLUSION

AML is a malignancy in need of new treatment alternatives, especially for patients unfit, ineligible for intensive chemotherapy. PARP inhibitors are targeted therapeutics for cancer that disrupt dysfunctional DNA damage response. Acute leukaemias with a special mutational landscape might be sensitive to the combinations of PARP inhibitors and cytotoxic molecules. NPM1 mutations are linked to dysfunctions in the DNA damage response. Therefore, we investigated whether NPM1‐positive AML cells are sensible to PARP inhibitors combined with chemotherapy agents. Our results show that possibly DNMT3A‐NPM1 mutated AML is as sensible to combinations of PARP inhibitors plus anthracyclines, but not to the combination of PARP inhibitors and hypomethylating agents, at least in a pre‐clinical setting.

## AUTHOR CONTRIBUTIONS

**Sabina Iluta:** Data curation (equal); Formal analysis (equal). **Sergiu Pasca:** Investigation (equal). **Grigore Gafencu:** Formal analysis (equal). **Ancuta Jurj:** Formal analysis (equal). **Andreea Terec:** Data curation (equal). **Patric Teodorescu:** Investigation (equal). **Cristina Selicean:** Investigation (equal). **Ciprian Jitaru:** Data curation (equal). **Alexandra Preda:** Investigation (equal); Methodology (equal); Resources (equal). **Diana Cenariu:** Investigation (equal); Methodology (equal). **Catalin Constantinescu:** Investigation (equal); Methodology (equal). **Maria Iordache:** Formal analysis (equal). **Bogdan Tigu:** Investigation (equal). **Raluca Munteanu:** Investigation (equal). **Richard Feder:** Formal analysis (equal). **Delia Dima:** Funding acquisition (equal). **Mihnea Zdrenghea:** Methodology (equal). **Diana Gulei:** Investigation (equal). **Tudor Eliade Ciuleanu:** Investigation (equal). **Ciprian Tomuleasa:** Investigation (equal); Validation (equal); Visualization (equal).

## Supporting information

Table S1

Table S2

Table S3

Table S4

Table S5

Table S6

## Data Availability

The data that support the findings of the study are available from the corresponding author upon reasonable request.
