# Supplementary material for: Azacytidine plus olaparib for relapsed acute myeloid leukaemia, ineligible for intensive chemotherapy, diagnosed with a synchronous malignancy
Source: J Cell Mol Med. 2021 Jun 16;25(13):6094–102. doi: 10.1111/jcmm.16513 (PMC8406486; doi:10.1111/jcmm.16513)
Supplement: Supplementary file 1 — Table S1 [file JCMM-25-6094-s004.docx]

| Cell Line | Timepoint | p value |
| --- | --- | --- |
| OCIAML3 | 48h | 0.315 |
| OCIAML3 | 72h | 0.707 |
| THP1 | 48h | 0.324 |
| THP1 | 72h | 0.493 |
