# Supplementary material for: Azacytidine plus olaparib for relapsed acute myeloid leukaemia, ineligible for intensive chemotherapy, diagnosed with a synchronous malignancy
Source: J Cell Mol Med. 2021 Jun 16;25(13):6094–102. doi: 10.1111/jcmm.16513 (PMC8406486; doi:10.1111/jcmm.16513)
Supplement: Supplementary file 2 — Table S2 [file JCMM-25-6094-s001.docx]

| Cell Line | Gene | p value |
| --- | --- | --- |
| OCIAML3 | PTEN | 0.576 |
| OCIAML3 | LIG4 | 0.161 |
| OCIAML3 | PARP1 | 0.52 |
| OCIAML3 | RAD5 | 0.265 |
| OCIAML3 | ATM | 0.018 |
| OCIAML3 | LIG3 | 0.068 |
| THP1 | PTEN | 0.053 |
| THP1 | LIG4 | 0.151 |
| THP1 | PARP1 | 0.033 |
| THP1 | RAD5 | 0.034 |
| THP1 | ATM | 0.309 |
| THP1 | LIG3 | 0.487 |
