# Supplementary material for: Azacytidine plus olaparib for relapsed acute myeloid leukaemia, ineligible for intensive chemotherapy, diagnosed with a synchronous malignancy
Source: J Cell Mol Med. 2021 Jun 16;25(13):6094–102. doi: 10.1111/jcmm.16513 (PMC8406486; doi:10.1111/jcmm.16513)
Supplement: Supplementary file 4 — Table S4 [file JCMM-25-6094-s006.docx]

| Cell Line | Treatment | p value |
| --- | --- | --- |
| OCIAML3 | Azacitidine | <0.0001 |
| OCIAML3 | Olaparib | 0.164 |
| THP1 | Azacitidine | <0.0001 |
| THP1 | Olaparib | <0.0001 |
