# Supplementary material for: Azacytidine plus olaparib for relapsed acute myeloid leukaemia, ineligible for intensive chemotherapy, diagnosed with a synchronous malignancy
Source: J Cell Mol Med. 2021 Jun 16;25(13):6094–102. doi: 10.1111/jcmm.16513 (PMC8406486; doi:10.1111/jcmm.16513)
Supplement: Supplementary file 6 — Table S6 [file JCMM-25-6094-s003.docx]

| Cell Line | Phase | p value |
| --- | --- | --- |
| OCIAML3 | G1 | <0.001 |
| OCIAML3 | S | <0.0001 |
| OCIAML3 | G2 | <0.0001 |
| OCIAML3 | G1 | 0.04 |
| OCIAML3 | S | 0.225 |
| OCIAML3 | G2 | 0.045 |
